# Loss of Plastid Developmental Genes Coincides With a Reversion to Monoplastidy in Hornworts

**DOI:** 10.3389/fpls.2022.863076

**Published:** 2022-03-14

**Authors:** Alexander I. MacLeod, Parth K. Raval, Simon Stockhorst, Michael R. Knopp, Eftychios Frangedakis, Sven B. Gould

**Affiliations:** ^1^Institute for Molecular Evolution, Heinrich-Heine-Universität Düsseldorf, Düsseldorf, Germany; ^2^Department of Plant Sciences, University of Cambridge, Cambridge, United Kingdom

**Keywords:** plastid evolution, bryophytes, hornworts, plant terrestrialization, plastid division

## Abstract

The first plastid evolved from an endosymbiotic cyanobacterium in the common ancestor of the Archaeplastida. The transformative steps from cyanobacterium to organelle included the transfer of control over developmental processes, a necessity for the host to orchestrate, for example, the fission of the organelle. The plastids of almost all embryophytes divide independently from nuclear division, leading to cells housing multiple plastids. Hornworts, however, are monoplastidic (or near-monoplastidic), and their photosynthetic organelles are a curious exception among embryophytes for reasons such as the occasional presence of pyrenoids. In this study, we screened genomic and transcriptomic data of eleven hornworts for components of plastid developmental pathways. We found intriguing differences among hornworts and specifically highlight that pathway components involved in regulating plastid development and biogenesis were differentially lost in this group of bryophytes. Our results also confirmed that hornworts underwent significant instances of gene loss, underpinning that the gene content of this group is significantly lower than other bryophytes and tracheophytes. In combination with ancestral state reconstruction, our data suggest that hornworts have reverted back to a monoplastidic phenotype due to the combined loss of two plastid division-associated genes, namely, ARC3 and FtsZ2.

## Introduction

Hornworts are a unique group of bryophytes, the monophyletic non-vascular sister lineage to all vascular land plants (Harris et al., 2020). The phylogenetic position of hornworts and their putative phenotypic resemblance to what one might consider to represent the last common ancestor of all land plants make them an attractive model for evo-devo studies linked to events such as plant terrestrialization (Frangedakis et al., 2020). Hornworts are the only group of land plants known to form a pyrenoid, a unique carbon-concentrating mechanism (CCM), otherwise common in algae; however, these CCMs are not present in all hornworts and are hence a poor taxonomic marker (Villarreal and Renner, 2012; Supplementary Figure 1).

Hornworts are one of the only groups of embryophytes that have not escaped the monoplastidic bottleneck. This is a phenomenon associated with plastid origin and the organelle’s integration into the host cell cycle, which constrains the majority of algae from possessing multiple plastids per cell (de Vries and Gould, 2018). One consequence is that the only plastids—of which there are five types in embryophytes (Jarvis and López-Juez, 2013)—hornwort cells house are chloroplasts, whose size and morphology vary across genera (Vaughn et al., 1992; Raven and Edwards, 2014; Li et al., 2017). To address the reason, we screened the genomes and annotated transcriptomes of ten hornwort species to identify the presence/absence of genes that play key roles in regulating plastid development, such as those involved in protein import into the chloroplast, thylakoid biogenesis, and chloroplast division (Jarvis and López-Juez, 2013). We highlight key differences between the developmental plastid biology of hornworts and other established model organisms in the terrestrial clade. Furthermore, we argue that the major changes in plastid biology, that not only coincided with major checkpoints in the evolutionary history of hornworts but also facilitated them, are a consequence of multiple instances of gene loss observed in this unique group of embryophytes.

## Hornworts Underwent Significant Instances of Gene Loss

We used the BUSCO version 5.2.2 (Manni et al., 2021) software to estimate the gene content of hornworts and compared them with other bryophyte and tracheophyte (vascular plant) outgroups (Supplementary Table 1). We found that the gene content of hornworts is significantly lower than tracheophytes and other bryophytes (ANOVA; *F* = 129.5; d.f. = 2,30; *p* < 0.001), thereby suggesting that hornwort diversification and speciation were accompanied by significant instances of gene loss (Supplementary Figure 2), even more than what is observed for bryophytes in general (Harris et al., 2021).

## Full Conservation of Translocon of the Outer Envelope of the Chloroplast but Only Partial Conservation of Translocon of the Inner Envelope of the Chloroplast in Hornwort Chloroplasts

**FIGURE 1:**
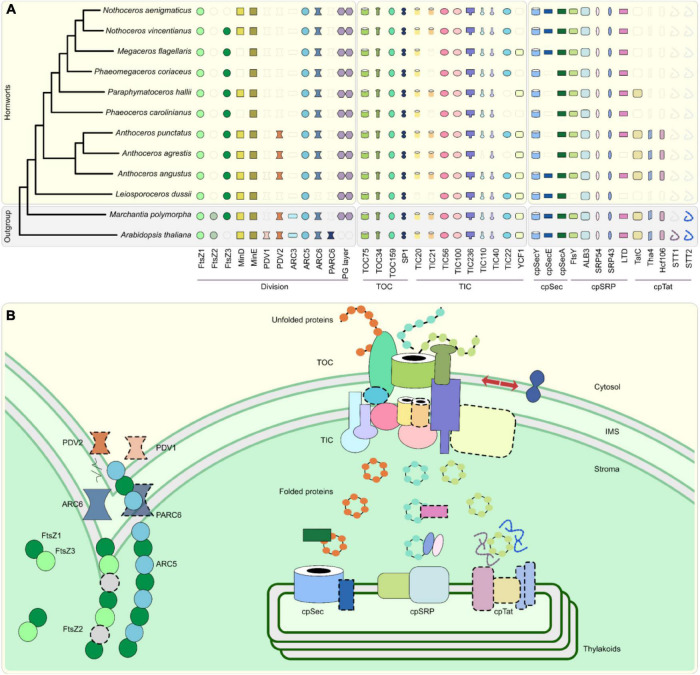
Plastid development and biogenesis in hornworts. **(A)** A presence/absence pattern (PAP) of various plastid developmental components that are sorted into three categories based on whether they are associated with plastid division (PD) and protein translocation across the plastid envelope *via* TOC/TIC or the thylakoid membrane. Transparent icons indicate that no gene could be identified. **(B)** A combined schematic representation of plastid development in embryophytes. Components that are absent from more than two hornworts in our surveyed taxa, or absent in this group altogether, are highlighted by dotted outlines. ARC, accumulation and regulation of chloroplasts; FtsZ, filamentous temperature Z; IMS, intermembrane space; Sec, secretory; SRP, signal recognition particle; Tat; twin arginine translocation; TOC/TIC, translocator of the outer/inner chloroplast membrane; PDV, plastid division. While ARC5 is absent from the *Anthoceros agrestis* Bonn ecotype, which we included in our OrthoFinder analyses as the representative for this species, our reciprocal best hit pipeline confirmed that it is present in the Oxford ecotype, with its gene ID being AagrOXF_evm.TU.utg000081l.174. A maximum likelihood (ML) tree was constructed *via* the IQ-TREE version 2.0.3 software (Minh et al., 2020), using an automated selection model, by concatenating single-copy chloroplast and mitochondrial markers from 65 different hornwort species, and three outgroups (Villarreal and Renner, 2012). Said sequences were aligned with MUSCLE in AliView (Edgar, 2004; Laarson, 2014). Gene trees for orthologs listed on the PAP were generated using the PhyML version 3.0 and IQ-TREE version 2.0.3 softwares using automated selection models (Guindon et al., 2010; Lefort et al., 2017). We used the SHOOT framework (Emms and Kelly, 2021) to extract orthologous sequences from across the Archaeplastida for said trees. We analyzed the genomes and transcriptomes of ten hornworts, along with the genomes of *Arabidopsis thaliana* and *Marchantia polymorpha*, to determine the presence of various components involved in plastid development (Lamesch et al., 2012; Bowman et al., 2017; Leebens-Mack et al., 2019; Li et al., 2020; Zhang et al., 2020; Supplementary Table 2). These orthology clusters (orthogroups) were identified using the OrthoFinder version 2.5.4 software (Emms and Kelly, 2015, 2019; Supplementary Table 2). To validate orthogroup presence/absence, we checked for reciprocal best hits using DIAMOND (Buchfink et al., 2015). Due to the difficulty in identifying orthologs for the import protein YCF1 in the Archaeplastida (de Vries et al., 2015), we employed a different strategy to identify orthologs for this gene. We extracted established YCF1 sequences from GenBank and UniProt and used them as queries for DIAMOND.

The vast majority of plastid proteins are encoded by the nuclear genome, and after their synthesis in the cytosol, are imported into the plastid by the translocon of the outer/inner envelope of the chloroplast (TOC/TIC) complex (Richardson and Schnell, 2020). Embryophytes have evolved the most sophisticated TOC/TIC complexes (Gould et al., 2008; Knopp et al., 2020) and our data confirm that the hornwort TOC complex is comprised of the same key proteins that are found in other embryophytes, mainly TOC75, TOC34, and TOC159 (Richardson and Schnell, 2020; Figure 1). The recycling of major TOC components is regulated by the RING-type ubiquitin E3 ligase SP1, which targets these proteins for proteasomal degradation (Ling et al., 2012; Figure 1B).

The TIC complex of embryophytes is comprised of a 1 MDa multimer that forms a pore that receives precursor proteins from the TOC complex in the intermembrane space (IMS) and finally mediates their passage to the stroma (Nakai, 2015a; Richardson and Schnell, 2020; Figure 1). The presence/absence of TOC/TIC components reveals no pattern with regard to mono-/polyplastidy or presence/absence of a pyrenoid (Figure 1A and Supplementary Figure 1). However, some TIC components appear to have undergone differential loss in some hornwort taxa (Figure 1A), most notably TIC21, TIC22, YCF1 (TIC214), and maybe even TIC20 in *Leiosporoceros dussii*. The latter species is the only member of our surveyed taxa that lacks a TIC20 ortholog (Figure 1A).

YCF1/TIC214, the only TOC/TIC component encoded by the plastid genome and unique to the green lineage, is absent in a significant number of hornworts (Figure 1A), such as in *Nothoceros aenigmaticus*, for which also the plastid genome is available (Villarreal et al., 2013).

## Differential Loss of an Ancient Thylakoid Developmental Pathway in Most Hornworts

Thylakoid proteomes contain the bulk of photosynthesis-related proteins of plant cells (Xu et al., 2021). After their import *via* TOC/TIC, thylakoid proteins are recognized and sorted *via* one of three main pathways, the components of which are predominantly derived from the cyanobacterial endosymbiont or inserted spontaneously (Xu et al., 2021; Figure 1).

The chloroplast secretory (cpSec) pathway is involved in importing unfolded proteins to the thylakoid lumen. Powered by the motor protein cpSecA, unfolded subunits pass through a pore formed by cpSecY and cpSecE (Xu et al., 2021; Figure 1B). While the presence/absence of cpSec components reveals no pattern with regard to mono-/polyplastidy or presence/absence of a pyrenoid, half of surveyed hornworts lack cpSecE orthologs, with this distribution not showing any unique phylogenetic pattern (Figure 1A and Supplementary Figure 1).

The chloroplast twin-arginine translocation (cpTat) pathway can import folded proteins and is powered by the thylakoid’s proton motive force (PMF; Xu et al., 2021). In those hornworts, for which we identified the cpTat pathway, it is comprised of three proteins, namely, Tha4, TatC, and Hcf106 (Figure 1). Precursor proteins initially bind to a TatC-Hcf106 complex. Tha4 is subsequently recruited *via* the action of the PMF, undergoing a conformational change, leading to the passage of the precursor protein (Xu et al., 2021; Figure 1B). The presence/absence of cpTat components reveals no pattern with regard to mono-/polyplastidy or presence/absence of a pyrenoid; however, the cpTat pathway seems only to be encoded by the Anthocerotaceae, having been differentially lost in other hornwort families (Figure 1A).

The third main pathway involved in sorting proteins for thylakoid biogenesis is the chloroplast signal recognition particle (cpSRP) pathway. This translocation complex is involved in targeting specifically light harvesting complex proteins (LHCPs) to the thylakoid membrane (Xu et al., 2021; Figure 1B). LHCP integration is initiated when a rudimentary LHCP is transferred from the TIC translocon to the SRP43/SRP54 complex by the LTD protein. Subsequently, this SRP43/SRP54 complex binds to the FtsY receptor. GTP hydrolysis results in LHCP integration *via* the action of the ALB3 integral translocase (Xu et al., 2021; Figure 1B). Our results suggest that the cpSRP pathway is ubiquitous in all hornworts, as the core components of this pathway are present in the vast majority of our surveyed taxa; therefore, presence/absence of cpSRP components reveals no pattern with regard to mono-/polyplastidy or presence/absence of a pyrenoid. However, FtsY is absent in *L. dussii*, and LTD is absent in both *Anthoceros angustus* and *L. dussii*.

## Loss of Plastid Division Components Coincides With Monoplastidy in Hornworts

Plastid division in bryophytes is achieved by three components, namely, the outer and inner rings and most likely the peptidoglycan (PG) layer (Figure 1). The inner division ring (Z-ring) is comprised of FtsZ1, FtsZ2, and FtsZ3, while the outer division ring comprises ARC5 and FtsZ3 (Osteryoung and Pyke, 2014; Grosche and Rensing, 2017; Figure 1B). Z-ring and outer ring synchronization are achieved *via* an interplay of ARC6 and PDV2 (Osteryoung and Pyke, 2014). The PG layer is a relic of the chloroplast’s cyanobacterial past, and it might be relevant in regulating chloroplast division in bryophytes and streptophyte algae (Hirano et al., 2016; Grosche and Rensing, 2017).

**FIGURE 2:**
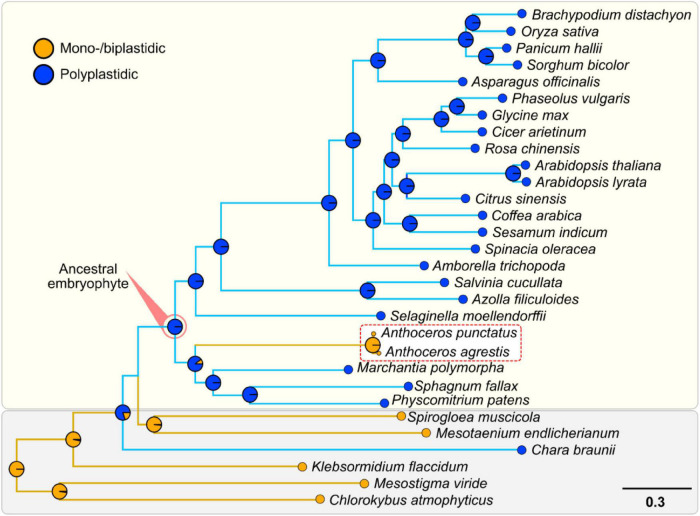
Support for the polyplastidic nature of the ancestral embryophyte and monoplastidic nature of the ancestral hornwort. Pie charts at the nodes display estimates of the probabilities for the plastidic phenotype of the respective most recent common ancestors (MRCAs). Hornworts are highlighted with a white box and a red dotted line. A robust ML species phylogeny of the green lineage was constructed *via* the IQ-TREE version 2.0.3 (Minh et al., 2020), using an automated selection model, by concatenating several housekeeping genes identified with DIAMOND in 34 different streptophytes, seven chlorophytes, and one glaucophyte (Supplementary Tables 3, 4; Buchfink et al., 2015). We used a reciprocal best hit pipeline with DIAMOND (Buchfink et al., 2015), to analyze the genomes of 34 different streptophytes to determine the presence and absence of orthologs involved in plastid division, to estimate the presence/absence of ARC3 and FtsZ2 at various nodes on our tree (Supplementary Table 5). Subsequent ASRs were undertaken using the ape function from the Phytools package (Revell, 2012).

Hornworts appear to have differentially lost *both* ARC3 and FtsZ2 (Figure 1A). This differential loss correlates with this group of bryophytes reverting back to a monoplastidic, or near-monoplastidic, phenotype (Figure 2 and Supplementary Figures 3, 4; Villarreal and Renner, 2012; Raven and Edwards, 2014; Li et al., 2017). Indeed, previous studies have shown that generating individual gene mutant lines of *ARC3* and *FtsZ2* in *A. thaliana* and the moss *Physcomitrium patens* causes fewer plastids (in the case of *arc3* mutants) or one giant plastid per cell (in the case of *ftsz2* mutants) (Pyke and Leech, 1992; Martin et al., 2009). ARC3 is part of the FtsZ family and unites an FtsZ domain with a C-terminal MORN domain (Zhang et al., 2013).

## Discussion

It is evident that hornwort—and bryophyte—emergence and diversification were accompanied by major instances of gene loss (Harris et al., 2021). Our results reinforce this hypothesis, specifically highlighting that the combined loss of certain genes may be responsible for the unique plastid phenotype observed in this group.

The absence of TIC20 in *L. dussii* could be the result of a transcriptome annotation and coverage issues (Cheon et al., 2020), since TIC20 is hypothesized to be a universal protein across the green lineage (Kalanon and McFadden, 2008; de Vries et al., 2015). Should this not be the case, then, maybe YCF1/TIC214 and TIC100 can compensate for TIC20’s absence in a unique manner. Some putative absences of YCF1/TIC214 could also be the result of assembly and/or annotation errors; however, the gene was lost without question in grasses, too (de Vries et al., 2015; Nakai, 2015b). The loss of this import protein does not lead to the loss of the entire import capacity (Bölter and Soll, 2017) and raises the question whether there is a functional, causative correlation between the loss of YCF1/TIC214 across these diverse embryophyte groups.

Considering cpSecE only plays an accessory role in protein translocation by tiling and rotating cpSecY’s N-terminal half, its absence in some hornworts indicates that it might not be detrimental to the function of the cpSec pathway (Figure 1B; Park et al., 2014). If the cpTat pathway is indeed absent in most hornwort families, then this raises the question on how the thylakoids import folded proteins. Furthermore, all hornworts appear to lack STT proteins (Figure 1A), which mediate liquid-liquid phase transitions (LLPTs), allowing for more efficient sorting of cpTat substrates (Figure 1; Ouyang et al., 2020). cpTat-related LLPTs hence appear absent in hornworts or are regulated otherwise. The differential loss of FtsY and LTD in *L. dussii* could be a consequence of this species potentially losing TIC20, with this core TIC component being a key LTD interaction partner (Ouyang et al., 2011).

We found that the chloroplasts of all surveyed hornworts possess all the enzymes necessary for PG layer biosynthesis (Figure 1A), hinting toward a conserved function similar to that in the moss *P. patens* (Hirano et al., 2016). While ARC3 orthologs are absent in some polyplastidic seedless plants (such as *P. patens* and the lycophyte *Selaginella moellendorffii*), these species then possess orthologs for FtsZ2, which might compensate its loss to some degree (Rensing et al., 2008; Albert et al., 2011; Zhang et al., 2013). This is further supported by an ancestral state reconstruction analysis that demonstrates that the ancestral embryophyte possessed both ARC3 and FtsZ2 and was polyplastidic, the opposite of which is true for the ancestral hornwort (Figure 2 and Supplementary Figures 3, 4). We predict that the loss of both genes contributed to the monoplastidic nature of hornworts and that reintroducing them might induce a polyplastidic phenotype.

## Conclusion and Outlook

We suggest that a consequence of some of plastid-related gene losses, including the combined loss of FtsZ2 and ARC3, resulted in hornworts reverting back to a monoplastidic phenotype, which the embryophyte ancestor was able to escape. If the knockout of ARC3 and FtsZ2 in *A. thaliana* and *P. patens* results in monoplastidic phenotypes, could one reverse evolution by expressing ARC3 and/or FtsZ2 in a hornwort? We anticipate our study to be a starting point for further experiments aimed at deconstructing bryophyte plastid biology and reconstructing new evolutionary hypotheses for outstanding questions in this topic. Next to exploring the monoplastidic bottleneck, hornworts might be able to shed new light on the import of folded proteins into the thylakoid of non-Anthocerotaceae hornworts, or the consequences of a potential TIC20 loss in *L. dussii* and the detailed function of YCF1; which, like all grasses, some hornworts appear to have lost.

## Data Availability Statement

The datasets presented in this study can be found in online repositories. The names of the repository/repositories and accession number(s) can be found in the article/Supplementary Material.

## Author Contributions

AM undertook the phylogenomic analyses, with help from SS and MK. AM, SG, PR, and EF wrote the manuscript. All authors contributed to the article and approved the submitted version.

## Conflict of Interest

The authors declare that the research was conducted in the absence of any commercial or financial relationships that could be construed as a potential conflict of interest.

## Publisher’s Note

All claims expressed in this article are solely those of the authors and do not necessarily represent those of their affiliated organizations, or those of the publisher, the editors and the reviewers. Any product that may be evaluated in this article, or claim that may be made by its manufacturer, is not guaranteed or endorsed by the publisher.
