# Supplementary material for: Loss of Plastid Developmental Genes Coincides With a Reversion to Monoplastidy in Hornworts
Source: Front Plant Sci. 2022 Mar 14;13:863076. doi: 10.3389/fpls.2022.863076 (PMC8964177; doi:10.3389/fpls.2022.863076)
Supplement: Supplementary file 2 [file Data_Sheet_2.docx]

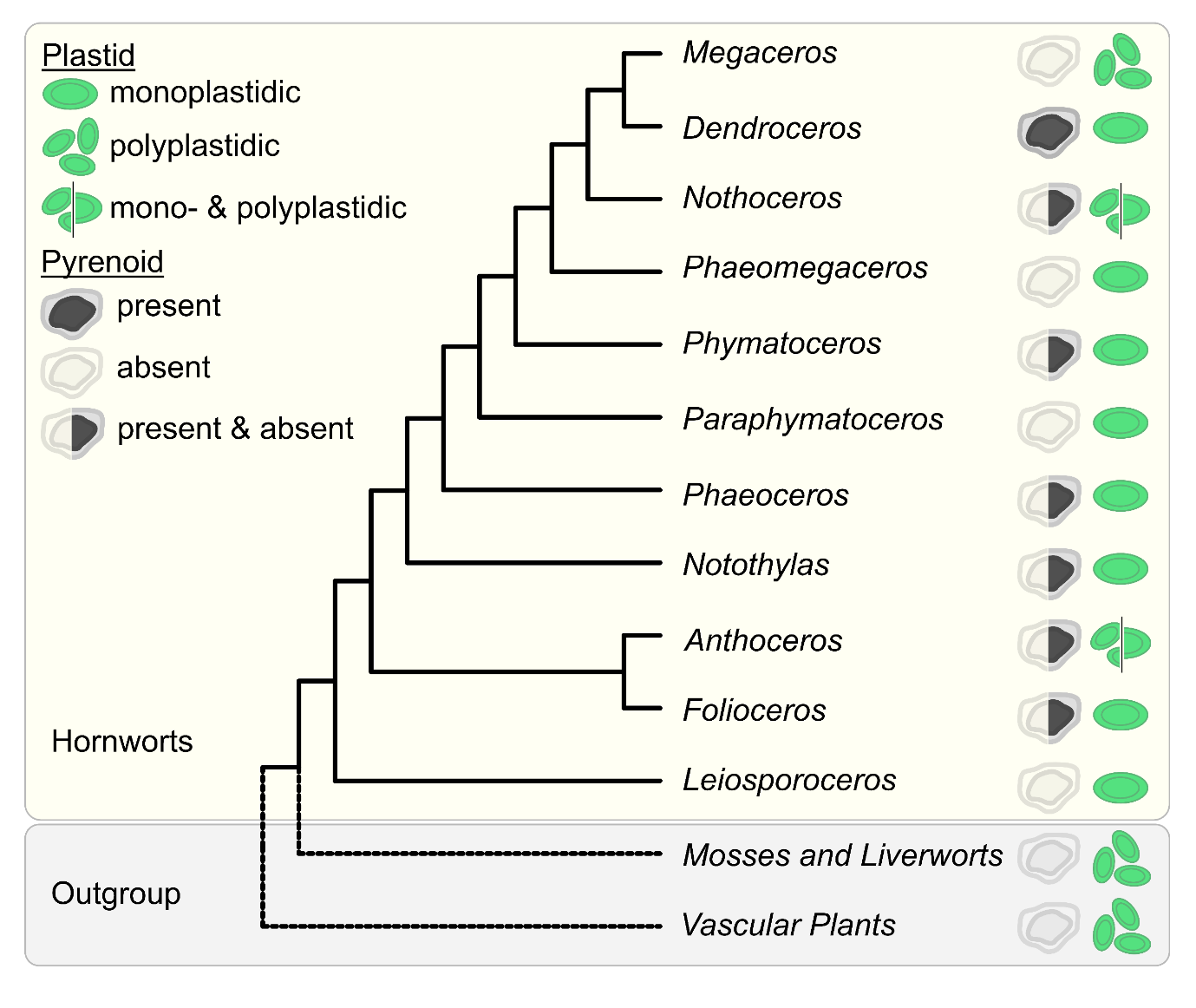


***Supplementary Figure S1*. Maximum likelihood (ML) phylogeny of the main eleven hornwort families.** Pyrenoidal and thallus/gametophyte plastidic phenotypes for the genera are indicated, based on Vaughan *et al*. (1992), Li *et al.* (2017), Villarreal *et al.* (2012) and Raven & Edwards (2014). Outgroups are highlighted by the dotted lines.


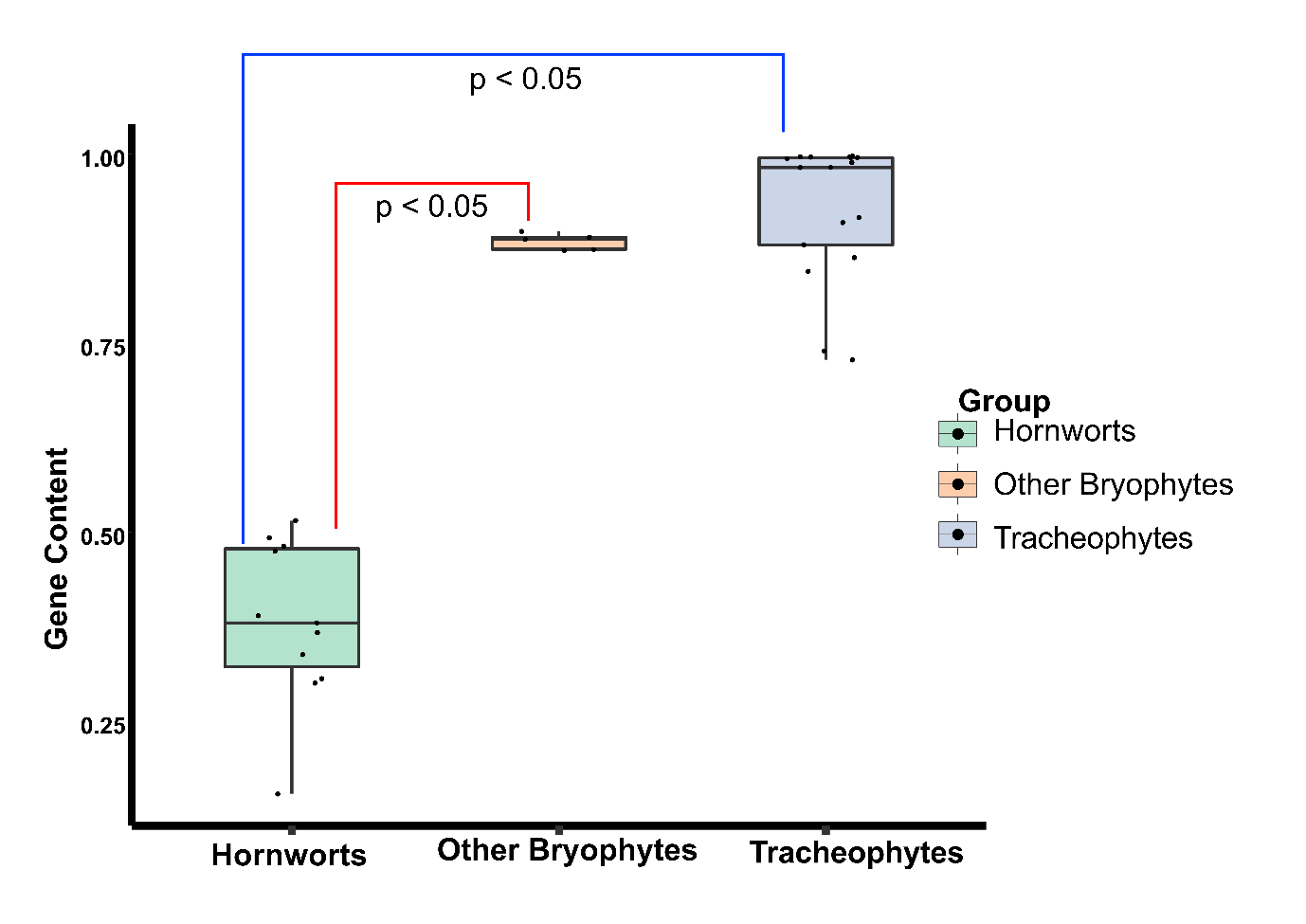


***Supplementary Figure S2*. Boxplot confirming significant instances of gene loss in hornworts.** Gene content was estimated by using BUSCO version 5.2.2 by using parameters specific to embryophytes (Manni et al., 2021).


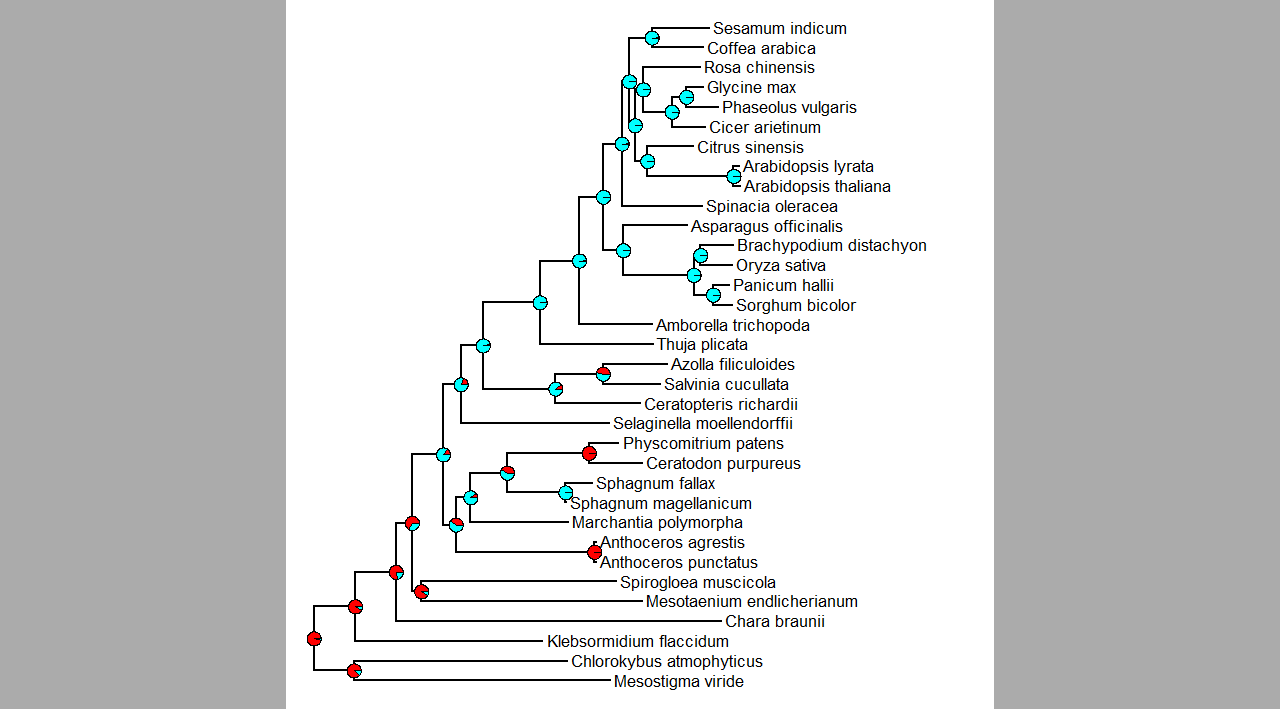


***Supplementary Figure S3***. ASR analysis showing the probability for the presence/

absence of ARC3 at various nodes in the tree. Presence is indicated by cyan; absence by

red.


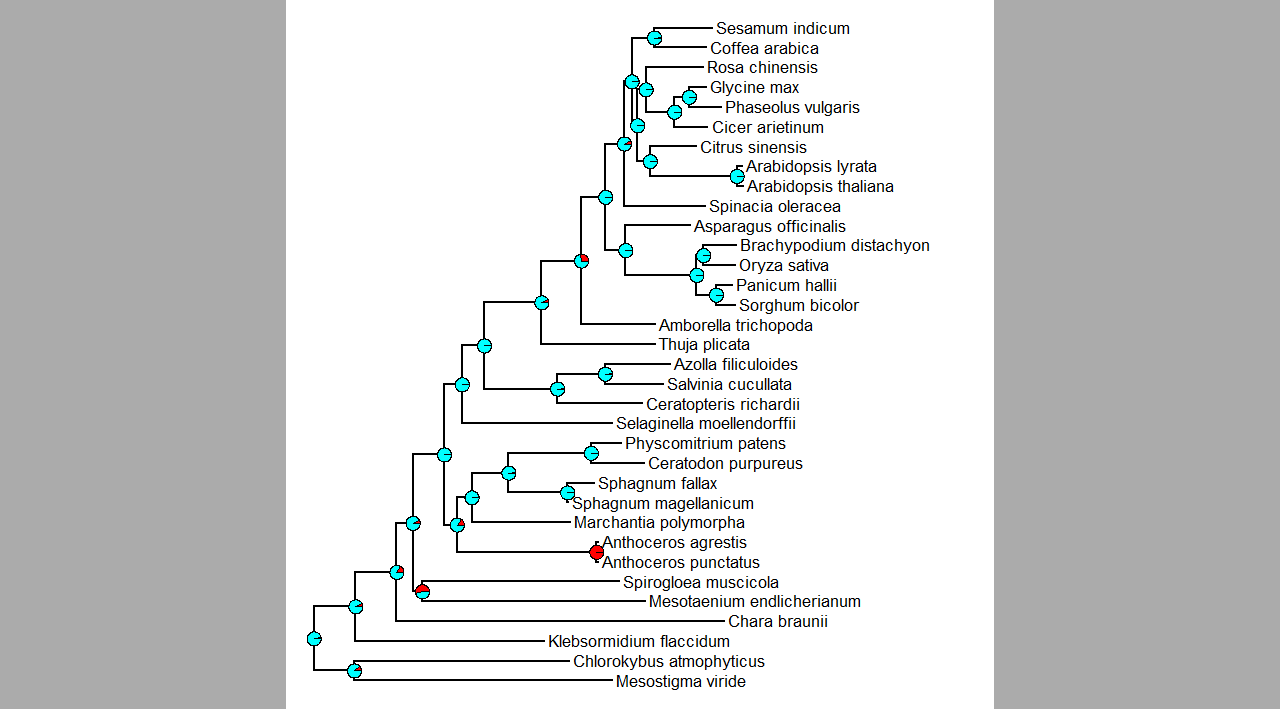


***Supplementary Figure S4***. ASR analysis showing the probability for the presence/

absence of FtsZ2 at various nodes in the tree. Presence is indicated by cyan; absence by

red.
